# Alkali-Activated Cements for TES Materials in Buildings’ Envelops Formulated With Glass Cullet Recycling Waste and Microencapsulated Phase Change Materials

**DOI:** 10.3390/ma12132144

**Published:** 2019-07-03

**Authors:** Jessica Giro-Paloma, Camila Barreneche, Alex Maldonado-Alameda, Miquel Royo, Joan Formosa, Ana Inés Fernández, Josep M. Chimenos

**Affiliations:** 1 Departament de Ciència de Materials i Química Física, Universitat de Barcelona, C/Martí i Franquès 1, 08028 Barcelona, Spain; 2 Birmingham Centre for Energy Storage & School of Chemical Engineering, University of Birmingham, Birmingham B15 2TT, UK

**Keywords:** alkali activated cement, CSP, microencapsulated phase change material, thermal energy storage, buildings

## Abstract

Within the thermal energy storage field, one of the main challenges of this study is the development of new enhanced heat storage materials to be used in the building sector. The purpose of this study is the development of alkali-activated cements (AACs) with mechanical properties to store high amounts of heat. These AACs incorporate wastes from industrial glass process as well as microencapsulated phase change materials (mPCMs) to improve the thermal inertia of building walls, and accordingly respective energy savings. The research presented below consists of the exhaustive characterization of different AACs formulated from some waste generated during the proper management of municipal waste used as precursor. In this case study, AACs were formulated with the waste generated during the recycling of glass cullet, namely ceramic, stone, and porcelain (CSP), which is embedding a mPCM. The addition of mPCM was used as thermal energy storage (TES) material. The mechanical properties were also evaluated in order to test the feasibility of the use of the new formulated materials as a passive TES system. The results showed that the AAC obtained from CSP (precursors) mixed with mPCMs to obtain a thermal regulator material to be implemented in building walls was reached successfully. The material developed was resistant enough to perform as insulating panels. The formulated materials had high storage capacity depending on the PCM content. The durability of the mPCM shell was studied in contact with alkaline medium (NaOH 4 M) and no degradation was confirmed. Moreover, the higher the content of mPCM, the lower the mechanical properties expected, due to the porosity increments with mPCM incorporation in the formulations.

## 1. Introduction

Nowadays, one of the most important challenges in society is the proper supply, availability, and efficient management of energy. Currently, social policies are focused in reducing CO_2_ emissions and controlling energy demand, which is exponentially growing. Aimed to control these issues, several constraints have been established for the growing use of fossil fuels and the misuse of energy. In this way, some possible solutions rely on research into new sources of renewable energy and in the study of new suitable materials, which are more sustainable and energy-efficient [1]. In this regard, in the last few years, the use of new recycled materials in construction field has been studied, which are more sustainable and allow for more efficient energy use. The main profit of these investigations are materials developed as insulating resources in buildings, materials that decrease the necessity (or increase the efficiency) of heating, ventilating and air conditioning (HVAC), and materials that need less energy to be manufactured and spread less CO_2_ emissions to the environment.

The building sector consumes 34% of the total energy consumption in Europe, and the residential buildings subsector is the one of the biggest consumers, accounting for around 80% of energy consumption [2]. Thereby, the final energy consumed for space conditioning (heating and cooling) accounts up to 66% of total energy used in the sector. Energy consumed by other heat uses as domestic hot water or water for heating and cooking accounts for 22% of total energy consumption in the sector. These are the reasons that make thermal energy storage a key enabling technology, as defined by the EU in the past few years [3].

Thermal energy storage (TES) is a technology that allows the storage and release of heat. There are several ways to store thermal energy in buildings as thermal comfort regulation [4], conditioning systems [5], façades [6], domestic hot water [7], and many others. The main proposed materials to store thermal energy in buildings are phase change materials (PCMs). Prof. Cabeza et al. stated the most used PCM in the building sector in 2011 [8] and Navarro et al. benchmarked the available PCMs in the market for building purposes [9].

Indeed, PCMs are substances with high storage capacity. This means that when they melt or solidify at certain temperatures, they are capable of storing and releasing large amounts of energy. Heat is absorbed or released when the material changes state. PCMs are classified within the latent heat storage (LHS) category. The organic PCMs have low thermal conductivity, which means they have long time requirements to charge and discharge the storage systems. Moreover, since the PCMs are liquid during some periods, there are leakage issues that must be solved. Therefore, microencapsulation is the technical solution to address these two problems [10]. Most of the reported microencapsulated PCMs (mPCMs) consist of a polymeric shell containing the core PCM.

Alkali activated cements (AACs) are a subcategory of alkali activated materials (AAMs). AACs consist of compact and stable materials formed by the reaction of alkali hydroxide or alkali silicate activation with a solid powder precursor rich in aluminosilicates [11]. Then, AACs can be classified into three main categories: moderately calcium-rich cements (CASH, calcium aluminum silicate hydrate), low calcium cements (NASH, sodium aluminum silicate hydrate), and those others that are a combination of these aforementioned cements and contain both kinds of gels [12].

AACs are presented as an appropriate alternative to ordinary Portland cement (OPC) in the field of construction [13]. It is well known that the cement industry is one of the most polluting, being responsible for approximately 7% of CO_2_ emissions worldwide and a large consumer of primary energy (estimated at approximately 3% of global consumption). In this sense, the cement industry is facing the challenge of finding new ways to reduce its environmental impact by consuming less energy, and reducing emissions. Among these, the development of more sustainable alternative cements, like AAC, with less greenhouse gas emissions and capable of having waste incorporated into their formulations, will contribute to the EU’s strategic policies for 2050, where society is low in carbon and the efficient in the use of resources [14] is mandatory. There are numerous investigations searching for new AACs based on wastes such as: red mud [12,15], glass cullet [16], fly ash and glass cullet [17], fluorescent lamps [18], and others. It is important to consider the large number of factors that can influence AAC properties when using by-products [19], such as the chemical activator, post-fabrication curing regime, particle size distribution of source materials, aggressive environmental exposure on mechanical strength, physical properties, microstructures and durability properties.

Regarding the glass recycling industry, the main by-product obtained is the cullet, which refers to both broken glass and waste glass. Broken glass comes from rejections of industrial processes and it is called the internal cullet. Waste glass comes from pre-consumers and post-consumers and is called the external cullet. In external cullet, different contaminants are found: ceramic (C), stone (S), and porcelain (P), known as CSP. Previous works [20,21] showed that CSP is approximately composed by 8 wt.% of ceramics, stones, and porcelain, another 8 wt.% of metals, plastics and organic materials, and 84 wt.% is the remaining glass that has not been able to be classified by optical sorting equipment during recycling (bottle necks and bottoms). Therefore, the use of this CSP waste is interesting, because currently it is not used, but landfilled.

The main goal of this study was to find an AAC made from an admixture of the CSP precursors and mPCM (as storage material) and use it as a material for a TES passive system performing in building walls/ceilings. The durability of mPCM in alkaline media is a requirement to achieve useful formulations. Thus, a reactivity test is performed to evaluate the mPCM shell stability in alkaline pH, to prevent paraffin leakage from the microcapsule. Moreover, the CSP precursor was analyzed by particle size distribution (PSD), X-ray fluorescence (XRF), X-ray diffraction (XRD), Fourier transformed infrared spectroscopy (FT-IR), thermogravimetrical analysis (TGA), and helium pycnometer. In order to evaluate the use of these AACs for passive walls in non-structural building envelopes, the thermal conductivity was evaluated. The mPCM used in this study was deeply characterized in previous studies [22]. Finally, exhaustive characterizations of AACs were carried out including FT-IR, TGA, differential scanning calorimetry (DSC), and scanning electron microscopy (SEM).

In summary, the aim of this study was to develop a sustainable binder or AAC, considered a sustainable construction material, by including glass cullet waste in its formulation and mPCM to decrease the material flow of waste and improve energy savings in the building sector.

## 2. Materials and Methods

### 2.1. Materials

#### 2.1.1. CSP

During the glass recycling process, it is required to remove non-convenient materials contained in the residual flow to be recycled. These materials are non-glassy materials (ceramics and porcelain) which have been improperly deposited and collected, which severely interferes with glass recycling, and which the final content in recycled glass is strictly limited (20 g per ton). It is estimated that the amount of improper materials in the glass container can reach approximately 2 wt.% of the total collected amount [23], which includes polyethylene terephthalate (PET) containers, ceramics and porcelain (cups and plates), and some metal waste as the most common improper materials. Their elimination is relatively simple for metallic and plastic remains, by means of electromagnetic and mechanical separators, respectively. Nevertheless, in the case of ceramics and porcelain, their separation becomes a difficult issue that requires the use of optical sorting equipment, which allows discrimination of opaque fragments from transparent or translucent ones. However, up to 80% of CSP waste is glass since the optical sorting does not recognize translucent or opaque fragments of glass, the parts of bottles with labels, and the bottle necks and bottoms, resulting in their final destination being sent to the landfills.

In the present study, a representative sample of the integral fraction of CSP (without metals, nor plastic, and papers) provided by Daniel Rosas, S.A. (Barcelona, Spain), was used as a precursor in the formulation of AAC. Its chemical composition, determined by means of X-ray fluorescence (XRF, WDXRF, Axios PW 4400/40, Malvern Panalytical, Malvern, UK), is summarized in the Table 1. The loss of ignition amounts in the CSP sample is attributable to the environmental moisture retained in some materials, e.g. the paper of the labels located on the glass cullet.

Prior to its usage, the CSP mass percentage was evaluated (Table 2). Afterwards, it was cleaned with soap and water for 12 days, with the cleaning solution changed every day. Subsequently, the cleaned CSP was dried at 105 °C for 24 h, and was then quartered to obtain representative subsamples. 

The materials composition of the sample under study is shown in Table 3. A total amount of 1 kg was grinded and milled with a ball mill until reaching a particle size less than 100 μm.

#### 2.1.2. Microenscapsulated Phase Change Material (mPCM)

The mPCM used in this study was the Micronal^®^5008 from BASF^®^ (Germany), fully characterized in a previous study [10]. It is octadecane paraffin microencapsulated in an acrylic polymeric shell. The phase change temperature of Micronal^®^ 5008 is 23 °C and its main properties are listed in Table 4. The selection of this mPCM was based on its phase change temperature, which suits Mediterranean weather during most of year, as proved in previous studies [10].

Based on previous studies, the minimum amount of mPCM to be included is 10 wt.%, at least [24]. Therefore, this is the minimum amount of mPCM to be added to the formulations in this study.

#### 2.1.3. Alkali-Activated Cements

In order to determine the best curing method (dry or wet curing), several samples were prepared. It was observed that wet curing, unlike common cement, had a negative effect, since the samples obtained presented very poor setting and mechanical properties. On the other hand, with dry curing, it was possible to appreciate that, after three days, a compact solid was formed where the mPCM was uniformly distributed inside the cementitious matrix. Hence, dry curing was followed when preparing the different samples.

To prepare the formulations, the CSP was progressively poured into a NaOH solution for 2 min. Afterwards, the mPCM was added while the mixture was still fluid, mixing for two additional minutes. Later, in order to compact the paste and reduce the porosity, the mixtures were vibrated for 30 seconds using a vibrating table. Subsequently, the obtained pastes with proper workability were poured in molds and sealed in a plastic bag for three days. The bags were kept in a stove at 40 °C to facilitate curing and avoid the water loss. On the third day, the plastic bag was removed. After seven days, the samples were unmolded but remained inside the stove for three weeks.

Four different formulations were studied, where the unique variable factor was the mPCM amount (wt.%) with respect to the solid samples. Three replicates for each formulation were evaluated. Regarding the NaOH activator, 4 M NaOH was the concentration used for all the formulations, considering a 0.6 liquid-to-solid (L/S) ratio.

The formulations under study are listed in Table 5.

### 2.2. Characterization of the CSP, mPCM, and AAC

In order to properly characterize the CSP waste, several parameters have been evaluated. The particle size distribution (PSD) of the sample was measured by means of a Beckman Coulter Inc. LS 13.320 device (Atlanta, GA, USA) with a universal liquid module. Besides, with a Panalytical Philips (Amsterdam, The Netherlands) PW 2400 sequential X-ray equipped with the software UniQuant^®^ V5.0 spectrophotometer, XRF semi-quantitative analysis was performed to study the most stable oxide content of the CSP sample. The crystalline phases were determined by X-ray diffraction (XRD) analyses of the CSP raw material and the 0 wt.% mPCM formulation sample in a Bragg-Brentano Siemens (Munich, Germany) D-500 powder diffractometer with CuKα radiation with the X’Pert HighScore (Almedo, The Netherlands) with a 2006 software version. Furthermore, a thermogravimetric analysis (TGA) was performed to study the thermal stability of the AAC samples and to determine the decomposition temperature of the mPCM. ASDT-Q600 device from TA Instruments (New Castle, DE, USA) was used, in a 100 µL aluminum crucible with 50 mL·min^−1^ N_2_ flow between 50 °C–600 °C at a heating rate of 10 °C·min^−1^. From a chemical point of view, Fourier transformed infrared (FT-IR) spectroscopy was used to study the composition of the mPCM and the different AAC formulations by using a FT-IR Spectrum Two^TM^ from Perkin Elmer (Waltham, MA, USA, 400–4000 cm^−1^ working range) supported by a Dynascan™ interferometer and OpticsGuard^TM^ technology with attenuated total reflectance (ATR). This equipment was optimized with a wavelength range between 4000 cm^−1^ and 450 cm^−1^ and its standard spectral resolution was 0.5 cm^−1^.

As the curing process was performed in alkaline pH, the mPCM was previously tested in 4 M NaOH solution for six weeks. Afterwards, the mPCM sample was dried at 40 °C for 48 h and observed by scanning electron microscopy (SEM). In addition, the morphology of the mPCM embedded in the sustainable binders was characterized using an environmental scanning electron microscope (ESEM, Quanta 200 FEI, XTE 325/D8395, Hillsboro, OR, USA). The sample was stuck on the sample holder using double-sided tape and then the particle size and the morphology of the sample were observed. The working conditions were low vacuum and high voltage (15 kV), and the image was obtained by secondary electrons. Furthermore, the real density of the formulated AAC was measured with a Micrometrics^®^ (Norcross, GA, USA) Pycnometer AccuPyc 1330 at 24 °C.

The thermophysical properties of the samples under study were analyzed by differential scanning calorimetry (DSC). The temperature program applied was between 10 °C and 50 °C under a 0.5 °C ·min^−1^ heating rate. Around 25 mg of samples were analyzed inside 40 μL aluminum crucibles under 50 mL·min^−1^ N_2_ flow. The instrument used was a DSC822e Mettler Toledo (Greifensee, Switzerland).

Lastly, the compressive strength of the different formulated samples was determined by following the UNE-EN-196-1 standard, applying 20 kN load at 240 kg·s^−1^ speed until the sample fractured. The samples were cut and prepared using a diamond disk cutter 150 low speed diamond saw from MTI Corporation (Richmond, CA, USA) at 300 rpm.

## 3. Results and Discussion

### 3.1. mPCM: Alkali Resistance and Physico-Chemical Analysis

**Figure 1:**
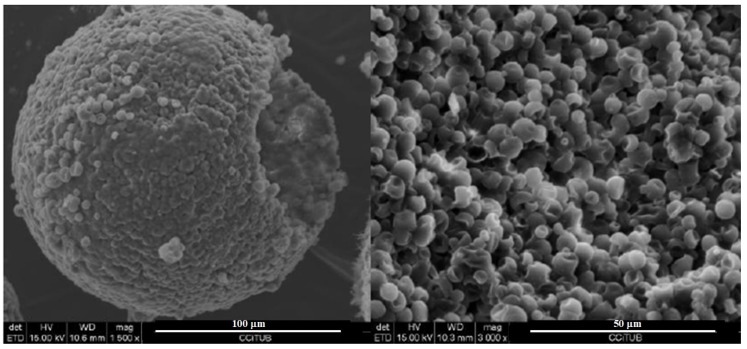
Scanning electron microscopy (SEM) micrographs of mPCM after six weeks in contact with 4 M NaOH.

The SEM images displayed in Figure 1 revealed that the mPCM sample under study was not degraded by the alkaline medium after six weeks. The left image shows the agglomeration of the microcapsules shown in the right picture. Both presented the same morphology as Micronal^®^ 5008 without an alkali treatment. Therefore, the microcapsules were basic-pH-resistant and stable when formulating the binders based in alkali-activated formulations.

### 3.2. CSP Physico-Chemical Characterization

**Figure 2:**
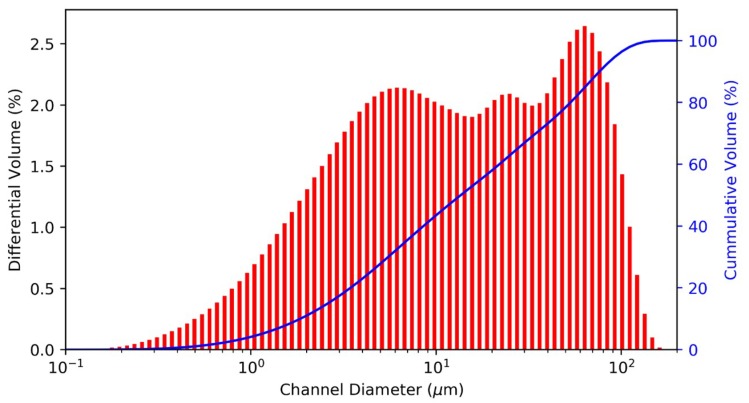
CSP particle size distribution.

In Figure 2 plots the particle size distribution of the milled CSP raw material, showing 90% of the particles have a diameter below 100 μm. Hence, this particle size provided high specific surface and reactivity of the precursor in the alkali medium.

### 3.3. AAC Characterization: Physico-Chemical, Thermal, and Mechanical

**Figure 3:**
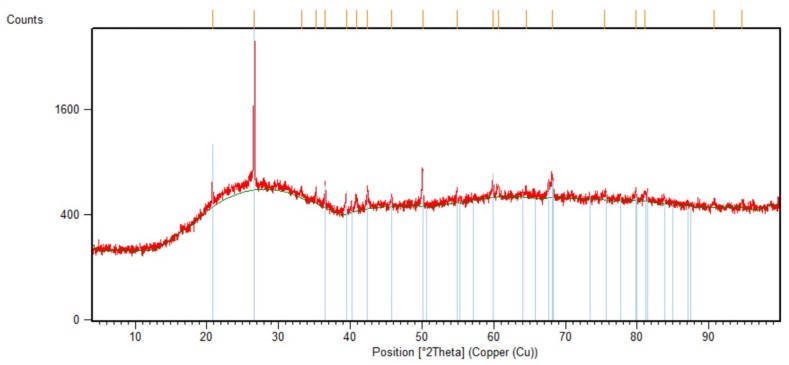
X-ray diffraction (XRD) results of the CSP used in this study.

XRD was performed (see Figure 3) on the AAC sample without mPCM; this spectrum presented an amorphous material as the baseline confirms, with few peaks corresponding to different crystalline phases. Moreover, the quartz (SiO_2_) and mullite (Al_6_Si_2_O_13_) spectra were highlighted in accordance with the XRD results.

**Figure 4:**
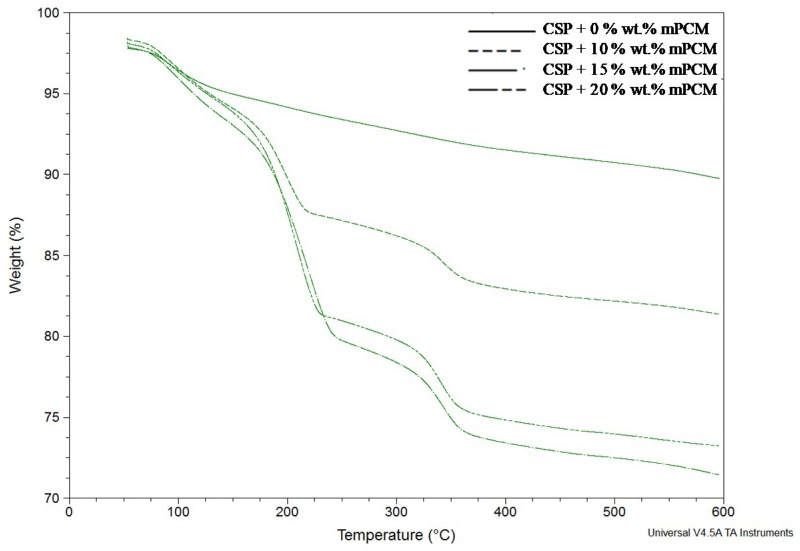
Thermogravimetric analysis (TGA) results comparison between the different formulations.

The comparison of the TGA results of the four AACs formulated is shown in Figure 4. The sample without mPCM showed a weight loss step due to water evaporation. The samples containing mPCM had three steps that corresponded to water evaporation (between 50 °C and 90 °C), PCM degradation (between 90 °C and 250 °C), and polymeric shell degradation (from 300 °C to the end of the TGA experiment). The degradation of the mPCM was in accordance with the results obtained by Giro-Paloma et al. in previous studies [22].

**Figure 5:**
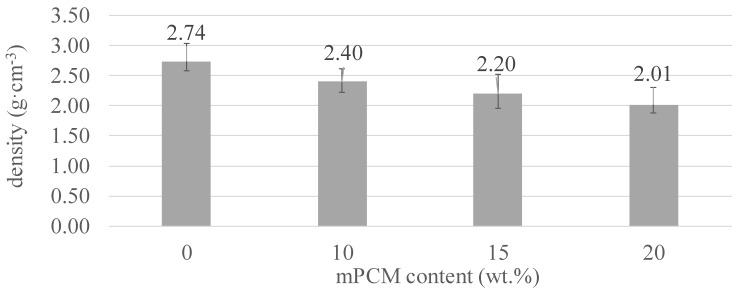
Density results for the different formulations.

The results of the real density analyzed by helium pycnometer for three specimens are shown in Figure 5. The error bar shows the standard deviation between the three tested specimens. The results showed the following trend: the higher the mPCM content, the lower the AAC’s real density, as expected by applying the mixture law [25], due to the lower density of the mPCM. On the other hand, an increment of the porosity was also observed when adding mPCM to ceramic materials, as stated in preceding studies by Serrano et al. [24]. Therefore, it should also be expected that a decrease in the bulk density of the AAC occurred.

The DSC results elucidated the heat storage capacity of the AAC formulated in this study. Table 6 lists the DSC results in terms of phase change enthalpy (ΔH_m_) and phase change temperature (*T_m_*), both during the melting process.

The *T_m_* obtained in all DSC analyses was similar and around 21.7 °C. The heat storage capacity was proportional to the *ΔH_m_*, independent of the mPCM content. Therefore, the DSC results of the storage capacity were in accordance with the mPCM content, and the storage capacity was higher than the material without mPCM. Hence, the thermal inertia increased when the AACs containing mPCM were applied. Accordingly, the AAC thermo-regulates the oscillation of the inner temperature of buildings, making the air conditioning system more efficient achieving proper energy savings, as measured by Castell et al. [26,27]. In this study, Dr. Castell studied cubicles containing 10 wt.% PCM in their facades and they achieved around 15%–17% energy savings in comparison with the analogue cubicles without PCM. Therefore, the material presented here is expected to produce at least 15% net energy savings when comparing the implementation of the same material without PCM, or even higher as the PCM content is two times that used in Dr. Castell et al. study.

**Figure 6:**
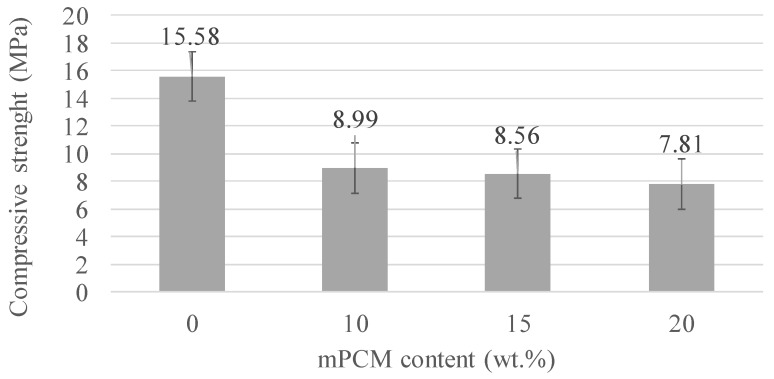
Compressive strength results for the different alkali-activated cement (AAC) samples.

As the AAC may be used as a TES passive system in non-structural building envelopes, their mechanical properties were evaluated (per triplicate). The compressive strength of the different formulations, depending on the mPCM content (wt.%), is shown in Figure 6, where the error bar is the standard deviation between the three tested specimens. As expected, the higher the mPCM content, the lower the mechanical resistance. This is because of the polymeric nature of the mPCM and its low mechanical properties, as well as the increasing porosity as the mPCM amount is increased [24].

## 4. Conclusions

The alkaline activated cements (AACs) or new sustainable binders formulated from secondary sources are an excellent alternative to OPC, since waste materials are revalorized in a new material life cycle, meaning more sustainable materials which reduce embodied energy and reduce the emission of CO_2_ and other greenhouse gases. The use of the CSP waste obtained during glass cullet management was appropriate to formulate the AAC. Therefore, this study proved that it was possible to formulate sustainable binders with CSP waste as precursor, combined with mPCM, to improve the thermal properties of constructive solution materials (from 7 kJ·kg^−1^ to 19 kJ·kg^−1^ when the mPCM content increased from 10 wt.% to 20 wt.%). Besides, it was demonstrated that the mPCM (Micronal^®^ 5008) used in this study is not damaged after six weeks of contact with alkali medium. These sustainable binders produce direct energy savings when applied in building envelopes (reducing the CO_2_ emissions during the in-use phase) and promote the reduction of materials to landfill. 

An exhaustive characterization of the CSP raw material, mPCM, and the different formulations was performed. The increasing mPCM content increased the porosity of the sustainable binders, and consequently the density (from 2.74 to 2.01 g·cm^−3^, 0 wt.% mPCM and 20 wt.% mPCM, respectively) and the mechanical properties diminished (from 16 MPa to 8 MPa, 0 wt.% mPCM and 20 wt.% mPCM, respectively). Nevertheless, the results of the mechanical strength of the AAC formulated with CSP demonstrated the potential and viability of these materials for use in non-structural construction fields compared to OPC mortars. Finally, the use of CSP as precursor to AAC is an interesting solution for its revalorization of waste as a secondary source.

## Figures and Tables

**Table 1:** Ceramic, stone, and porcelain (CSP) chemical characterization by means of X-ray fluorescence (XRF).

Components	SiO_2_	Na_2_O	CaO	Al_2_O_3_	MgO	K_2_O	Fe_2_O_3_	TiO_2_	LOI
**Amount (%)**	70.78	11.15	9.37	4.81	1.61	0.94	0.57	0.13	0.99

LOI: Lost of Ignition (900 °C).

**Table 2:** CSP mass percentage.

Size Fraction	Mass Percentage (%)
>16 mm	32.5
16–8 mm	59.0
8–4 mm	7.0
4–2 mm	1.0
2–0 mm	0.5

**Table 3:** Composition of waste glass.

Material	Amount (wt. %)
Glass (primary and secondary)	84.1
Porcelain	6.1
Ceramic	5.7
Stone	1.2
Polymer & paper	1.6
Metals	0.2
Organic	0.8
Non-classified	0.3

**Table 4:** Micronal^®^ 5008, microencapsulated phase change material (mPCM) properties list.

Name	Type	Melting Temperature	Storage Capacity	Latent Heat	Bulk Density
Micronal^®^ 5008	Powder	23 °C	135 kJ·kg^−1^	100 kJ·kg^−1^	300 kg·m^−3^

**Table 5:** Sample formulations under study.

Formulation	mPCM (wt. %)
1	0
2	10
3	15
4	20

**Table 6:** Differential scanning calorimetry (DSC) results for the formulations under study.

mPCM Content	*T_m_* (°C)	*ΔH_m_* (kJ·kg^−1^)
10 wt.% mPCM	21.6 ± 0.2	7 ± 2
15 wt.% mPCM	21.9 ± 0.2	14 ± 1
20 wt.% mPCM	21.8 ± 0.1	19 ± 1
